# Supplementary material for: The Physiological Functions of AbrB on Sporulation, Biofilm Formation and Carbon Source Utilization in Clostridium tyrobutyricum
Source: Bioengineering (Basel). 2022 Oct 19;9(10):575. doi: 10.3390/bioengineering9100575 (PMC9598496; doi:10.3390/bioengineering9100575)
Supplement: Supplementary file 1 [file bioengineering-09-00575-s001.zip › bioengineering-1957762-supplementary.pdf]

Supplementary Material

Supplementary Table S1. Strains, plasmids and primers used in this study

**Strains/Plasmids/Primers**	**Description**	**Source**
**Strains**		
E. coli CA434	Donor cells for conjugation transformation	[34]
Ct	ATCC 25755	[27]
pcloneEZ-P*lac*-repeat	Used for P*lac* promoter amplification	Stored in laboratory
pcloneEZ-repeat-	Used for terminator amplification	Stored in laboratory
Ct/*ΔabrB*	ATCC 25755 with pM3/*ΔabrB*	This study
Ct/*abrB*	ATCC 25755 with pM2/*abrB*	This study
Ct/*ΔabrB-abrB*	*Ct*/*ΔabrB* with pM2/*abrB*	This study
Ct/*Δspo0A*	ATCC 25755 with pM3/*Δspo0A*	This study
Ct/*ΔsigF*	ATCC 25755 with pM3/*ΔsigF*	This study
Ct/*ΔsigE*	ATCC 25755 with pM3/*ΔsigE*	This study
Ct/*ΔsigG*	ATCC 25755 with pM3/*ΔsigG*	This study
Ct/*ΔsigK*	ATCC 25755 with pM3/*ΔsigK*	This study
**Plasmids**		
pMTL82151	ColE1 ori; Cm^{R}; pBP1 ori; *TarJ*	[63]
pMTL82151-Em	ColE1 ori; Em^{R}; pBP1 ori; *TarJ*	Stored in laboratory
pMTL83151	ColE1 ori; Cm^{R}; pBP1 ori; *TarJ*	Stored in laboratory
pM3/*ΔabrB*	From pMTL83151; P*lac-ΔabrB*	This study
pM2/*abrB*	From pMTL82151-Em; P*cat*1-*abrB*	This study
pM3/*Δspo0A*	From pMTL83151; P*lac-Δspo0A*	This study
pM3/*ΔsigF*	From pMTL83151; P*lac-ΔsigF*	This study
pM3/*ΔsigE*	From pMTL83151; P*lac-ΔsigE*	This study
pM3/*ΔsigG*	From pMTL83151; P*lac-ΔsigG*	This study
pM3/*ΔsigK*	From pMTL83151; P*lac-ΔsigK*	This study
**Primers**		
P*cat*1-F	gaaacagctatgaccgcggccgcGTAGACTTTAAGGATGGAACCTTTGA	
Pro-F	TGAAGTACATCACCGACGAGCAAG	
Pro-R	TGCTGCAAGGCGATTAAGTTGGGT	
P*lac*-F	gaaacagctatgaccGCGGCCGCTTATATACTTGGTTTATTTACTTGATTAT	
P*lac*-repeat(*abrB*)-s-R	agatacatgctggttcatatttctttaataATTTAAATACATCTCATGTTAAGGTTC	
spacer(*abrB*)--pb-F	aatatgaaccagcatgtatcttctgGTTGAACCTTAACATGAGATGTATT	
spacer(*abrB*)--term-R	gaaatataggagctcATAAAAAAATTGTAGATAAAACTATTTTATA	
abrB-H1-F	ctacaatttttttatgagctcCTATATTTCCATCATATATAAAATCTGC	
abrB-H1-R	gtttttacagaagaaggtaccTTATTTTCCTCCCTAAGTTCCA	

Supplementary Table S1. *Cont.*

Strains/Plasmids/Primers	Description	Source
abrB-H2-F	ggaaaataaggtaccTTCTTCTGTAAAAACAGGCTATTT	
abrB-H2-R	tctccatggacgcgtgacgtcCGGCACCTAGAATTCAATCA	
abrB-conf-F	AAGCTCTCCTTAGATACTGATTTAATTATTT	
abrB-conf-R	CTACACAAACTAAATTAATAGCAGATTTAGAC	
P*cat*1(*abrB*)-R	tcttacaacacctgttgatttcatAAAAACCACCCTTTCATAAATTA	
abrB-F	aaagggtggtttttgcggccgcATGAAATCAACAGGTGTTGTAAGA	
abrB-R	tctccatggacgcgtgacgtcTTATCTTCCTTCTTTTAATTCATTTA	
Plac-repeat(*spo0A*)-s-R	taatagtagtgagagcaagaaaacggtatATTTAAATACATCTCATGTTAAGGTTC	
spacer(*spo0A*)-pb-F	ttcttgctctcactactattagctatatcaGTTGAACCTTAACATGAGATGTATTTAA	
spacer(*spo0A*)-term-R	gcttctaagtcctatgagctcATAAAAAAATTGTAGATAAAACTATTTTATAAA	
spo0A-H1-F	ttttttatgagctcATAGGACTTAGAAGCGAAATAAAAGAAG	
spo0A-H1-R	attgatatcactttatctagaATATTTTACTCCCCTTTTATTAATAAATTCC	
spo0A-H2-F	gtaaaatattctagaTAAAGTGATATCAATGGTTAACAGGTTT	
spo0A-H2-R	acgacggccagtgccaagcttGTTATCTGTGATTTCGGTATTCTATTATTC	
spo0A-conf-F	CCCAGTGCGGAGTATTTGGTA	
spo0A-conf-R	ATTATCGTGTAGTCCCTAAATTCGTG	
Plac-repeat(*sigF*)-s-R	tctatcaagagaactggtgctccatcatccATTTAAATACATCTCATGTTAAGGTTC	
spacer(*sigF*)-pb-F	agcaccagttctcttgatagataagGTTGAACCTTAACATGAGATGTATT	
spacer(*sigF*)-term-R	gtcatacaccgagctcATAAAAAAATTGTAGATAAAACTATTTTATA	
sigF-H1-F	ctacaatttttttatgagctcGGTGTATGACAGCGTTGAGC	
sigF-H1-R	acgaaacctacttatggatccATTATTCACCTAACTTAATGATTTAAAA	
sigF-H2-F	ggtgaataatggatccATAAGTAGGTTTCGTAACATAAAAATAT	
sigF-H2-R	tctccatggacgcgtgacgtcGCCTTCTGGAGTTGTAACTGA	
sigF-conf-F	GGATAGTTCAGGCATAGGTGTT	
sigF-conf-R	TTAACTCCCTACTACTCCGCTT	
Plac-repeat(*sigE*)-s-R	tgtacccactgatatcaaatcttctacatATTTAAATACATCTCATGTTAAGGTTC	
spacer(*sigE*)-pb-F	atttgatatcagtgggtacaataggGTTGAACCTTAACATGAGATGTATT	
spacer(*sigE*)-term-R	cctaagagccggtaccATAAAAAAATTGTAGATAAAACTATTTTATA	
sigE-H1-F	ctacaatttttttatggtaccGGCTCTTAGGCTCTATTATGATT	
sigE-H1-R	atactaaagacatgcggatccAATTTTCCTCCAATTAATCATAGT	
sigE-H2-F	aggaaaattggatccGCATGTCTTTAGTATAAAATATAGCAT	
sigE-H2-R	tctccatggacgcgtgacgtcCTCTAGCTTGAAGTGCCTTATAA	
sigE-conf-F	TTGCATATTTATAATGTATTCTATGCT	
sigE-conf-R	AATGATACTGGATCTTGAATTGC	
Plac-repeat(*sigG*)-s-R	attgcttttataagaccaacacatccgaccATTTAAATACATCTCATGTTAAGGTTC	
spacer(*sigG*)-pb-F	tgttggtcttataaaagcaatagatGTTGAACCTTAACATGAGATGTATT	

Supplementary Table S1. *Cont.*

Strains/Plasmids/Primers	Description	Source
spacer(*sigG*)-term-R	tatgttgccgagctcATAAAAAAATTGTAGATAAAACTATTTTATA	
sigG-H1-F	ctacaatttttttatgagctcGGCAACATATGCATCCAGA	
sigG-H1-R	tacaatataaatataggtaccGCAATCAGCCCCTTTAGAA	
sigG-H2-F	gctgattgcggtaccTATATTTATATTGTAAGTAATATTAATGGG	
sigG-H2-R	tctccatggacgcgtgacgtcGGGCATTTCACTTCTCTTCA	
sigG-conf-F	TACTGGAGTCAATGTAGAAGATTTG	
sigG-conf-R	ATAACTAATATAGGCAAGTCTTCAATT	
Plac-repeat(*sigK*)-s-R	ttccccggataagaatatttttttacaatgATTTAAATACATCTCATGTTAAGGTTC	
spacer(*sigK*)-pb-F	aaaatattcttatccggggaaggatGTTGAACCTTAACATGAGATGTATT	
spacer(*sigK*)-term-R	tcactactggagctcATAAAAAAATTGTAGATAAAACTATTTTATA	
sigK-H1-F	ctacaatttttttatgagctcCAGTAGTGAATTCAGGTATATATGTAAAA	
sig-H1-R	ttaatcttaagaataggtaccAGCTGCTCCTCCTAATAGTGC	
sigK-H2-F	ggagcagctggtaccTATTCTTAAGATTAAAAAATATATAAAAATTT	
sigK-H2-R	tctccatggacgcgtgacgtcCCACATCCAGTTGGTCCTG	
sigK-conf-F	TTGCATATTTATAATGTATTCTATGCT	
sigK-conf-R	TGTATCTTTACCTATTTCTCTTTGATT	

Note: Small letters are homologous sequences and capital letters are specific sequences. Underline marks are enzyme digestion sites

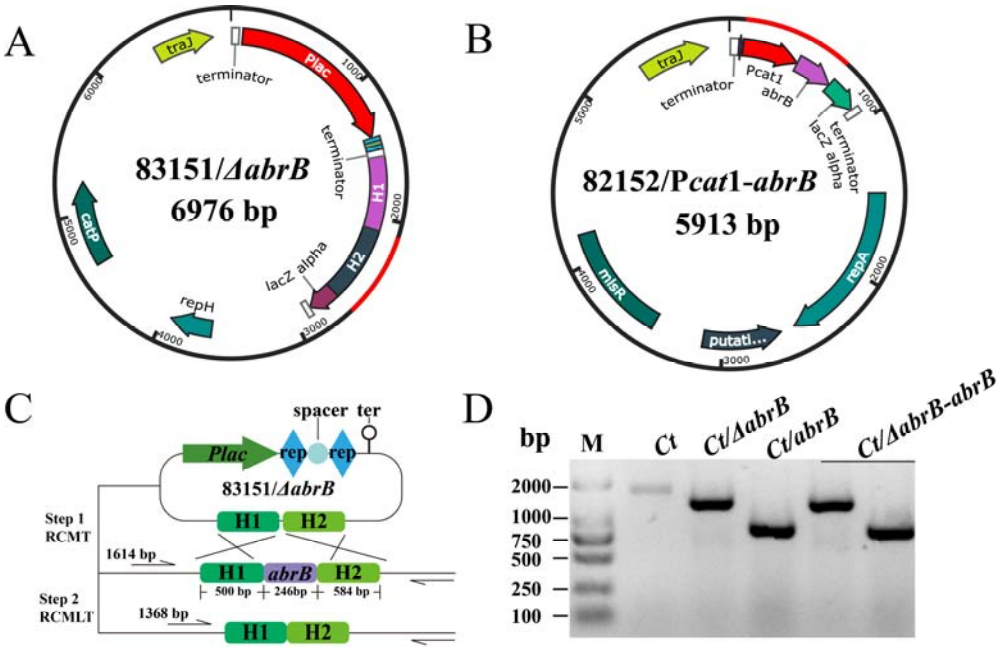

Supplementary Figure S1 Construction and verification of the *Ct/ΔabrB*, *Ct/abrB* and *Ct/ΔabrB-abrB* strains. (A) The *abrB* knockout plasmid; (B) The *abrB* overexpression plasmid; (C) Schematic illustrating the work flow of *abrB* gene deletion using the lactose inducible CRISPR-Cas system. (D) Gel electrophoresis verification of *abrB* mutant. M: Marker.

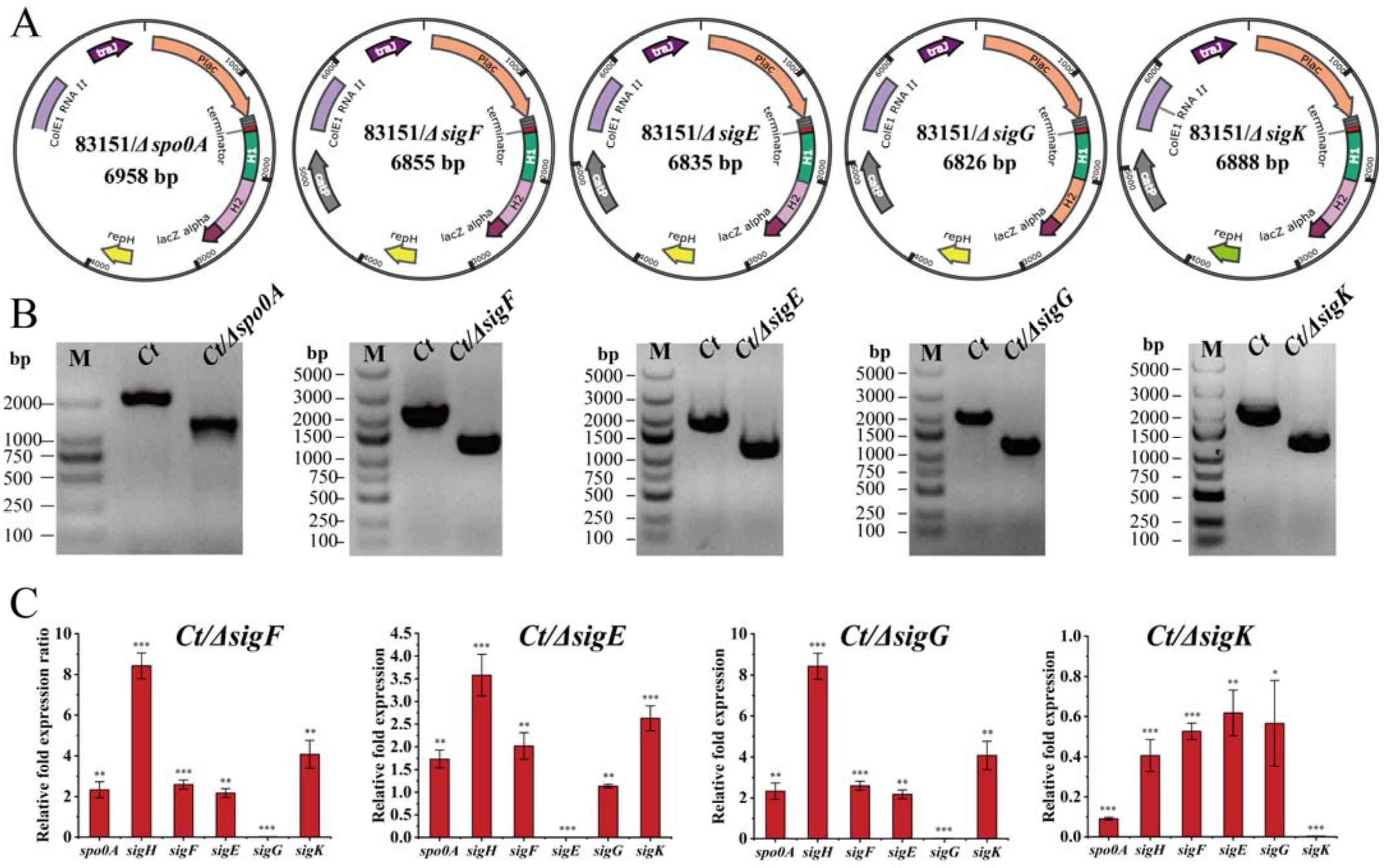

Supplementary Figure S2 Construction of *Ct/Δspo0A* and *Ct/Δsigma* strains. (A) Construction of the knockout plasmid of *spo0A* and sigma factors; (B) Gel electrophoresis verification of *spo0A* and sigma factors mutant; (C) qRT-PCR analysis

of genes related to sporulation transcription levels at 72 h. (ns, non-significant; *, $p<0.05$; **, $p<0.01$; ***, $p<0.001$, t-test).
